# Torsionator: Dihedral Parameterization and Conformer Search Made Easy

**DOI:** 10.1021/acs.jctc.6c01006

**Published:** 2026-09-09

**Authors:** Giorgia Brosio, Alice Triveri, Pietro Vidossich, Sergio Decherchi, Marco De Vivo

**Affiliations:** Laboratory of Molecular Modelling & Drug Discovery, 121451 Fondazione Istituto Italiano di Tecnologia, via Morego 30, Genoa 16163, Italy

## Abstract

Neural network potentials (NNPs), trained on quantum mechanical (QM) data, can deliver near QM-level accuracy while being much faster than QM calculations. In this work, we introduce a new software tool, Torsionator, which currently supports MACE, OBIWAN, and UMA NNPs for energy minimization of small molecules. Torsionator can analyze a set of selected conformers to parameterize the dihedral angles. These dihedral parameters can then be used for molecular mechanics calculations and simulations. We showcase Torsionator by performing dihedral scanning and parameterization of two representative antiviral compounds: favipiravir defluoro analog T1105 and emivirine. To thoroughly evaluate its robustness and general applicability, we benchmarked Torsionator using MACE on the TorsionNet500 dataset, comprising 500 chemically diverse small molecules with reference QM torsional profiles. These results indicate Torsionator as a practical, efficient, and scalable software tool for dihedral parameterization, enabling its routine integration into molecular simulation workflows.

## Graphical abstract

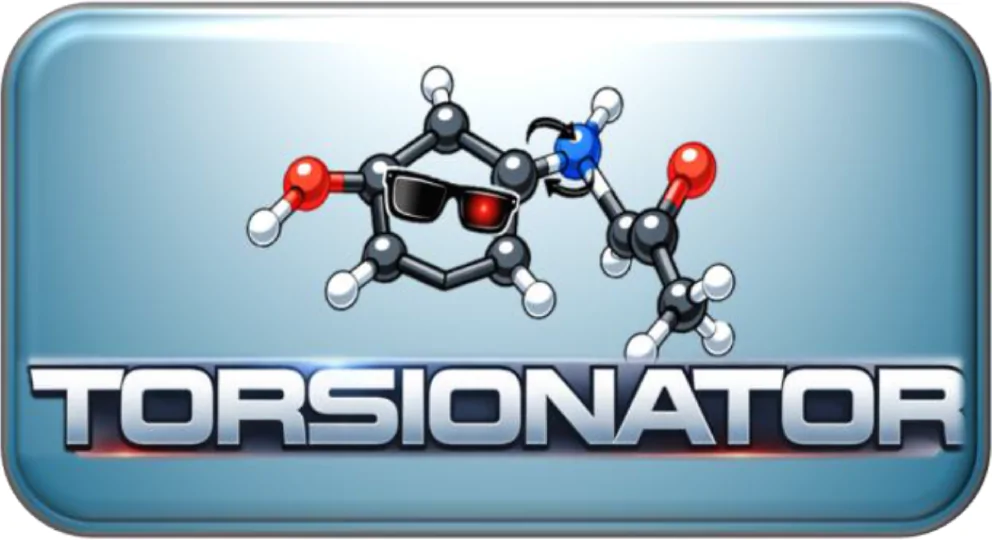

## Introduction

In recent years, artificial neural networks (ANNs) have been increasingly developed and used to construct so-called neural network potentials (NNPs).^1–3^ This growing activity and interest in NNPs are driven by their unique ability to combine near-quantum-mechanical (QM) accuracy, such as that of density functional theory (DFT), with fast computation (compared to costly QM calculations).^4,5^ Indeed, several popular neural network-based approaches capable of fitting QM potential energy surfaces have been developed, including for instance the Behler and Parrinello network,^6^ Deep Tensor Neural Network,^7^ ANI,^8^ OBIWAN,^9^ SchNet,^10^ PhysNet,^11^ MACE,^12,13^ AIMNet2,^14^ and UMA^15^ among others.^16^ This wealth of NNPs reflects the intense effort of the computational chemistry community to leverage machine learning algorithms for computations and molecular simulations.^17^

NNPs are based on a large, complex class of functions for approximating high-quality QM reference data, typically obtained from DFT or coupled-cluster calculations.^18–20^ NNPs are nonlinear regressors whose inputs are the atomic positions and chemical species of a given molecule. The input is encoded using descriptors or symmetry functions that are invariant to rotations, translations, and permutations, ensuring physical consistency. Equivariance is enforced in some architectures (e.g., MACE). These neural networks typically fall into two architectural families: graph-based and feed-forward. Graph neural network (GNN) models, such as MACE and SchNet, represent the molecular system as a graph in which atoms are nodes and bonds are edges equipped with feature vectors.^21^ Feed-forward NNPs, such as ANI, OBIWAN, and PhysNet, encode the local atomic environment through fixed, physics-inspired descriptors, typically atom-centered symmetry functions^22^ or radial basis functions,^11^ in a feature preprocessing step that does not rely on an explicit graph representation, yet still embeds the desired physical invariances by construction.^23,24^ All this generates an output consisting of the energy and, by derivation, forces of a given molecule.^6^

Force-field parameters are critical in molecular mechanics and atomistic molecular dynamics, particularly in drug discovery, where accurate predictions of conformational preferences and the energetics of small-molecule compounds can significantly impact their binding modes and affinity estimates for its target.^25,26^ Among the various components of a force field, the dihedral (torsional) term is particularly sensitive, as it governs molecular flexibility and the relative population of conformational states. The general form of the torsional energy term in molecular mechanics simulations is

1 $$V\left(\phi \right)=\underset{n\in S}{\sum }K_{n}\left[1+\cos\left(n\phi -\delta_{n}\right)\right]$$

Here, *K*_*n*_ is the force constant (Fourier coefficients), *n* the integer periodicity belonging to the set *S*, *δ*_*n*_ the phase offset, and ϕ the dihedral angle. Inadequate or poorly tuned dihedral parameters can result in inaccurate energy barriers, incorrect conformer distributions, and flawed predictions of key molecular properties such as free energy. This challenge is amplified in drug-like molecules, which often feature flexible scaffolds and torsional motifs that are frequently not well represented in standard force fields.^27,28^

Attempts to improve the description of dihedral energies in force fields led to an increase in the number of atom-type definitions required to specialize torsional terms.^29^ To limit atom typing while providing broad coverage of organic chemical space, the Generalized Amber Force Field (GAFF)^30,31^ uses heuristic rules to derive new parameters from known ones based on atom similarity. An alternative strategy consists in reparameterizing *ad hoc* the torsional terms of the molecule under investigation. The procedure for doing this task consists in performing a torsional scan with a high level of theory (usually DFT or MP2) and fitting the molecular mechanics Fourier coefficients to reproduce the higher-level energy profile.^30–32^ This approach is for instance automated by OpenFF BespokeFit^33^ to derive molecule-specific torsion parameters. To reduce the cost of computing the reference torsional energy profile, the use of NNPs has been advocated. Indeed, NNPs such as those in the ANI family have been shown to outperform classical force fields on drug-relevant torsional profiles^8,34^ and have been used to develop tools such as Parameterize^35^ and ParametrizANI.^36^ Recently, end-to-end frameworks such as Espaloma^38,39^ and Grappa^40^ use neural networks to assign all parameters, including torsions, directly from the molecular structure. These approaches can thus be grouped into two broad families: methods that compute an explicit torsional energy profile and subsequently fit classical parameters^33,35,36^ and methods that predict force-field parameters directly from the molecular graph in a single inference step.^38,40^

In this work, we introduce Torsionator, a software tool that leverages NNPs to perform dihedral scans of small organic molecules. Torsionator provides an integrated workflow that includes: (i) structure optimization; (ii) torsional energy scans; (iii) selection of suitable conformer(s) for scanning; (iv) derivation of molecular mechanics torsional parameters; and (v) conformational analysis of small molecules. Torsionator is open-source software and currently tailored to the Amber Force Field. It features a multi-conformer scan (MCS) procedure that constructs the torsional profile as the lower envelope over several conformer scans, strongly mitigating the typical steric-clash and scan-hysteresis artifacts encountered in single-conformer approaches. For ease of deployment, we deliver Torsionator as a software container (i.e., Apptainer). Torsionator employs molecular structures in several common formats as input and produces an Amber^41^ compatible *frcmod* file as output, enabling seamless integration into standard topology-building workflows. Torsionator interfaces with NNPs through the Atomic Simulation Environment API (ASE)^42^ ensuring generality and facilitating future extensibility. Another key feature of Torsionator is support for multiple NNPs, including MACE (MACE-OFF23 medium model), OBIWAN, and UMA-small. These NNPs enable the estimation of conformational energy in the gas phase, consistent with standard practice for small-molecule parameterization. Long-range effects are substantially neglected by the models currently employed.^32,33,36^

## Methods

### Workflow Description and Available Options

Torsionator currently supports three machine learning potentials: MACE,^12,13^ UMA,^15^ and OBIWAN.^9^ They are accessed via the Atomic Simulation Environment (ASE)^42^ library, which handles geometry optimizations and relaxed potential energy scans. While MACE and UMA provide a native ASE interface, a custom ASE calculator for OBIWAN was developed in this work. This custom interface takes atomic configurations from ASE and returns the energy and forces computed by OBIWAN (the necessary unit conversion is handled internally by the calculator). Since MACE and OBIWAN were trained exclusively on neutral molecules, whereas UMA was also trained on charged species, the tool warns the user to use UMA whenever the system has a non-zero net charge (and spin multiplicity different than 1, when needed; see Supporting Information for examples of charged molecules).

The workflow adopted in Torsionator is shown in Figure 1. An input file in PDB, SDF, MOL2, MOL, XYZ format and user-defined flags are passed to the software, which then provides the coordinates and corresponding energies of the generated geometries as output, together with an Amber-compatible force field parameter file (*frcmod* format). A *workflow.lo*g file stores information about the execution of each step and any error that may occur.

**Figure 1:**
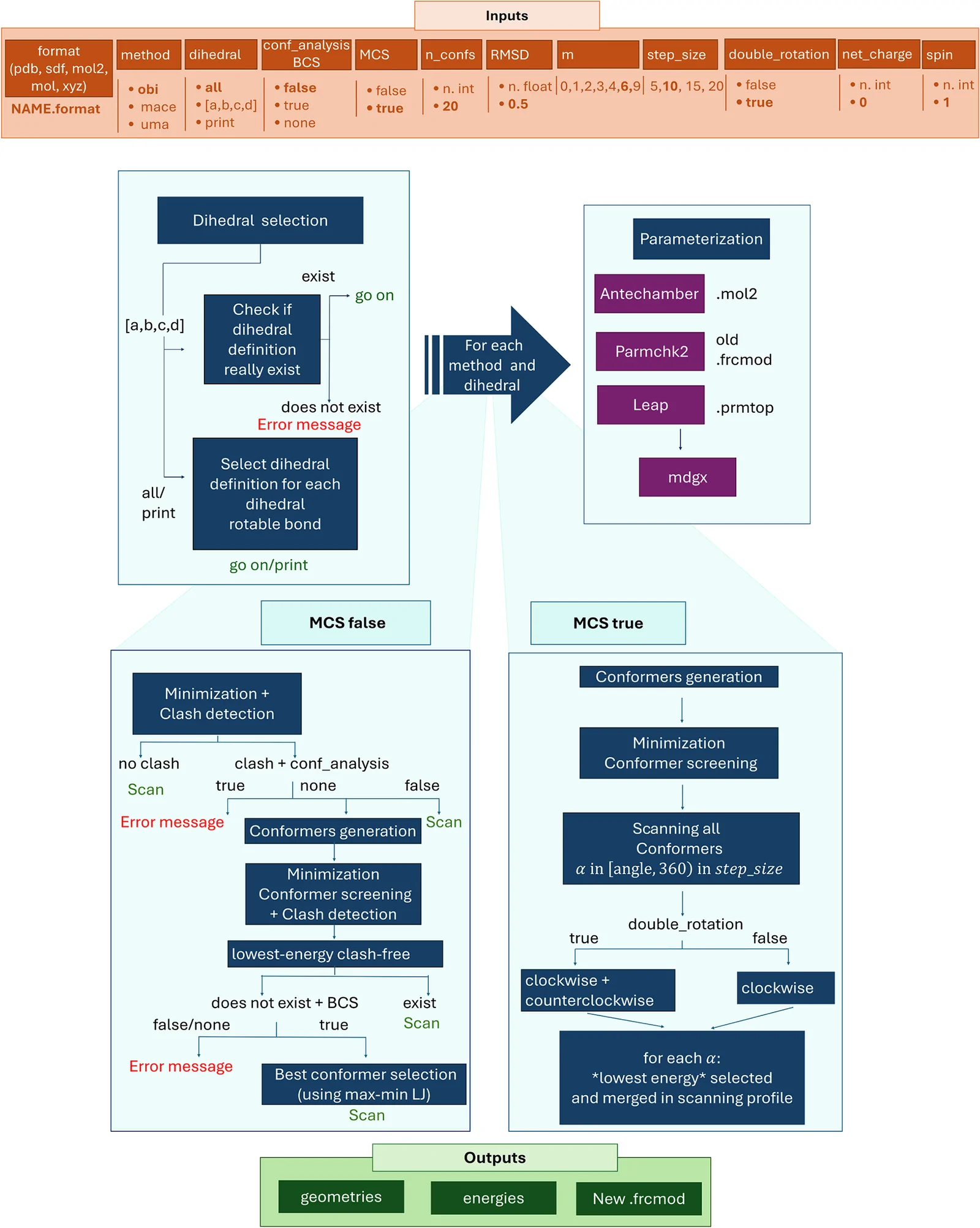
Starting from an input structure (supported formats are PDB, SDF, MOL2, MOL, XYZ) Torsionator searches for all rotatable bonds. In bold, in the input section, the default configuration parameters are reported. If “print” is requested, the unique definition of each dihedral angle detected is printed in the terminal (and saved in the workflow.log file), and the program terminates. If “all” or a specific dihedral is selected, the software proceeds as per the other blue insets in the picture. The molecule is minimized (via OBI/MACE/UMA); clashes are detected and optionally resolved via conformer generation followed by a constrained dihedral scan. Conformers can be used in two different ways: BCS (bottom-right subplot) or MCS (bottom-left subplot). The resulting energies and scan geometries are used for parameterization (blue insert) and generation of the corresponding *frcmod* Amber file. Parameterization is done using the mdgx program, which performs a least-squares fit of the torsional Fourier series coefficients against reference energies and structures provided by Torsionator’s dihedral scanning procedure. In the top red subplot, the input included options, and on the bottom green subplot, the output.

Torsionator employs the open-source cheminformatics toolkit RDKit^43^ to identify all rotatable bonds. A bond is classified as rotatable if it is a single, non-ring bond connecting two heavy atoms, each bearing at least one heavy substituent. Bonds involving terminal hydrogen atoms or terminal methyl groups are excluded. Each valid torsion is represented as a zero-based index quadruplet [a, b, c, d] where b–c defines the rotatable bond. Users can automatically process all detected torsions, print the list of available torsions, or specify a particular torsion for analysis. If a user-defined dihedral is not present in the structure, the program raises an error. More than one torsional term may be associated with a given rotatable bond; usually, parameterization of a single term is sufficient to correct the molecular mechanics behavior. Torsionator has three distinct modes to scan the selected dihedral angle: (i) using the single structure provided by the user; (ii) using a single structure selected from an ensemble of conformers (**Best Conformer Scan, BCS -** *conf_analysis=true* and *BCS=true)*; (iii) using an ensemble of conformers (**Multi Conformer Scan, MCS -** *MCS=true*). The rational resides in the possibility that unfavorable steric interactions may occur during torsional scanning, resulting in artificial energy spikes and discontinuities that compromise the torsional profile and subsequent parameter fitting.

After optimizing the input geometry (see Supporting Information for details), Torsionator evaluates steric feasibility via a preliminary rotation. Given the torsion [a, b, c, d], the molecular graph is partitioned into two fragments: a “left group” containing atoms *-b and a “right group” containing atoms c-* (where * indicates the attached atoms at any graph distance). Pairwise distances between atoms belonging to these groups are monitored during rotation. A steric clash is detected when the distance between any atom pair falls below the sum of their van der Waals radii, minus a buffer of 0.3 Å.

If no clashes are detected, a relaxed dihedral scan is performed. Starting from the initial dihedral angle (obtained via ASE’s *get_dihedral­()* method), the torsion is sampled using increments defined by *scan_step* (10° by default). At each step, the dihedral is constrained using ASE’s FixInternals, while relaxing the other degrees of freedom. The optimized structures and corresponding energies are recorded. Resulting geometries are stored in PDB and XYZ format and subsequently converted into NetCDF trajectory files using *cpptraj*.

If steric clashes are detected, the user may either proceed with the scan on the current structure (*conf_analysis=false*) or trigger a conformational analysis *(conf_analysis=true*). In the latter case, an ensemble of conformers (default: *n_confs=20*) is generated from the minimized structure using the distance geometry ETKDG algorithm^44^ available in RDKit.^43^ These conformers are optimized with the MMFF94s force field, discarding similar structures based on a user-defined RMSD threshold (default is 0.5 Å). The original input structure is included in the ensemble. Each conformer is optimized and evaluated for steric feasibility using the above clash-detection procedure, and the clash-free conformer with the lowest NNP energy is selected for subsequent scanning. If no conformer in the ensemble is free of steric clashes, the user may either terminate the workflow (*BCS=false*) or proceed with a suboptimal conformer (*BCS=true*). In the latter case, conformers are ranked based on their steric behavior during a preliminary torsional scan. Specifically, for each conformer, the Lennard-Jones (LJ) interaction energy between “left” *-b and “right” c-* groups along the scan is computed. The selected conformer is the one that minimizes this maximum LJ value, i.e., the conformer that exhibits the least severe steric clashes across the torsional pathway.

The MCS workflow, inspired by the procedure used to develop TorsionNet,^37^ uses all generated conformers to produce a single energy profile. A relaxed torsional scan is performed for each conformer in the ensemble. At each sampled dihedral angle, the lowest-energy structure across all conformers is retained, yielding a merged profile. The MCS approach is particularly advantageous for generating smooth profiles for parameter fitting when steric clashes are expected, such as in flexible molecules with multiple rotatable bonds. The resulting MCS profile does not constitute a continuous conformational pathway. From a physicochemical point of view, this corresponds to estimating a global profile that carries information across an entire set of conformations. Hence, employing such a profile represents a conformation-guided regularization technique for obtaining parameters.

Torsionator saves a plot showing the energy of every conformer in the ensemble as a function of the torsional angle, together with the lower-envelope profile identified by the Multi-Conformer Scan (MCS) method and subsequently used for the torsional parameterization (see Figure S8). To further reduce hysteretic effects in torsional scans, MCS optionally performs rotations in both clockwise and counterclockwise directions (*double_rotation=true*, default) to enrich the set of energy data points that are merged into a single profile (see Figure S9). In contrast, for molecules that do not exhibit steric clashes during rotation, a single-conformer scan (or BCS mode) provides a more directly interpretable representation of the torsional pathway, corresponding to the barrier along a single conformational transition. In the absence of clashes, the BCS mode is thus preferable.

### Molecular Mechanics Torsional Profile

Torsionator provides a comparison of the NNP torsional energy profile to the Generalized Amber Force Field (GAFF2). To this aim, AmberTools programs *antechamber*, *parmchk2,* and *LEaP* are used to build the topology and model system for the input molecule. Using this parameterization, the GAFF2 torsional energy profile is obtained by single-point energy evaluation on the same set of geometries generating the NNP profile.

### Reparameterization of GAFF2 Torsional Terms

The NNP torsional energy profile and corresponding optimized geometries, together with the GAFF2 parameters and topology, are used to reparameterize the force-field torsional terms of the selected dihedral angle using the *mdgx* ^45^ program from AmberTools. Mdgx performs a least-squares fit of the torsional Fourier series coefficients against reference energies and structures provided by Torsionator’s dihedral scanning procedure. All required files, including format conversion and input file, are generated by Torsionator. The set S of terms included in the Fourier expansion (eq 1) is specified in Table S2 by the parameter m, where the default m = 6 means using n = 1, 2, 3, 4, 6. Alternatively, users can decide to use a different number of terms (passing a value to the *m* flag) or retaining the original GAFF2 functional form (which is encoded by *m* = *0*). All terms introduced in addition to those present in GAFF2 are initialized with zero amplitude. The fitting in *mdgx* can be regularized introducing a restraint (whose strength is set by the flag *hrst)*, which penalizes departing from the initial value of the force constant. Rather than selecting a single *hrst* value a priori, Torsionator spans several *hrst* values and creates a family of results, hence dealing with *hrst* as a model selection hyperparameter. For each *hrst* value, *mdgx* produces a candidate force field parameter file, each of which is used to produce a torsional energy profile. The parameters minimizing the RMSE (Root Mean Square Error) between the reference NNP energies and the force field energies are selected for output. When −*dihedral=all* is selected a single *mdgx* parameterization is performed for each regularization strength (*hrst*), simultaneously fitting all dihedral terms using the NNP-derived energies and geometries from the individual torsion scans, with the optimal *hrst* selected as the one minimizing the worst-case RMSE across all dihedrals.

Torsionator is written in Python and available at https://github.com/giobros/torsionator.git. It is also made available via an Apptainer container to easy its portability and reproducibility.

## Results

To demonstrate Torsionator’s performance with increasing complexity, we apply the workflow to three case studies: first, a small-molecule drug with a single rotatable bond; second, a molecule with multiple rotatable bonds where best-conformer scanning and multi-conformer scanning prove advantageous; and third, the entire TorsionNet500 dataset of 500 drug-like fragments.^37^

### Torsional Scan and Reparameterization of a Dihedral without Steric Hindrance

To exemplify this setting, we used the molecule T1105 (Figure 2A), a defluorinated analog of favipiravir (T-705), a broad-spectrum antiviral that acts as a purine-mimicking nucleotide analog. Its mutagenic effect arises from its ability to base-pair with both cytidine and uracil.^46^ Indeed, rotation of the carboxamide group relative to the aromatic core exposes different H-bonding patterns, allowing either base pairing. Thus, molecular modeling studies addressing the base-pairing properties of T1105 (and, similarly, favipiravir) should accurately account for the rotation of the carboxamide group. As shown in Figure 2C, GAFF2 overestimates the rotational barrier relative to quantum-chemical results, thereby hindering the observation of conformational interchange during MD simulations.

**Figure 2:**
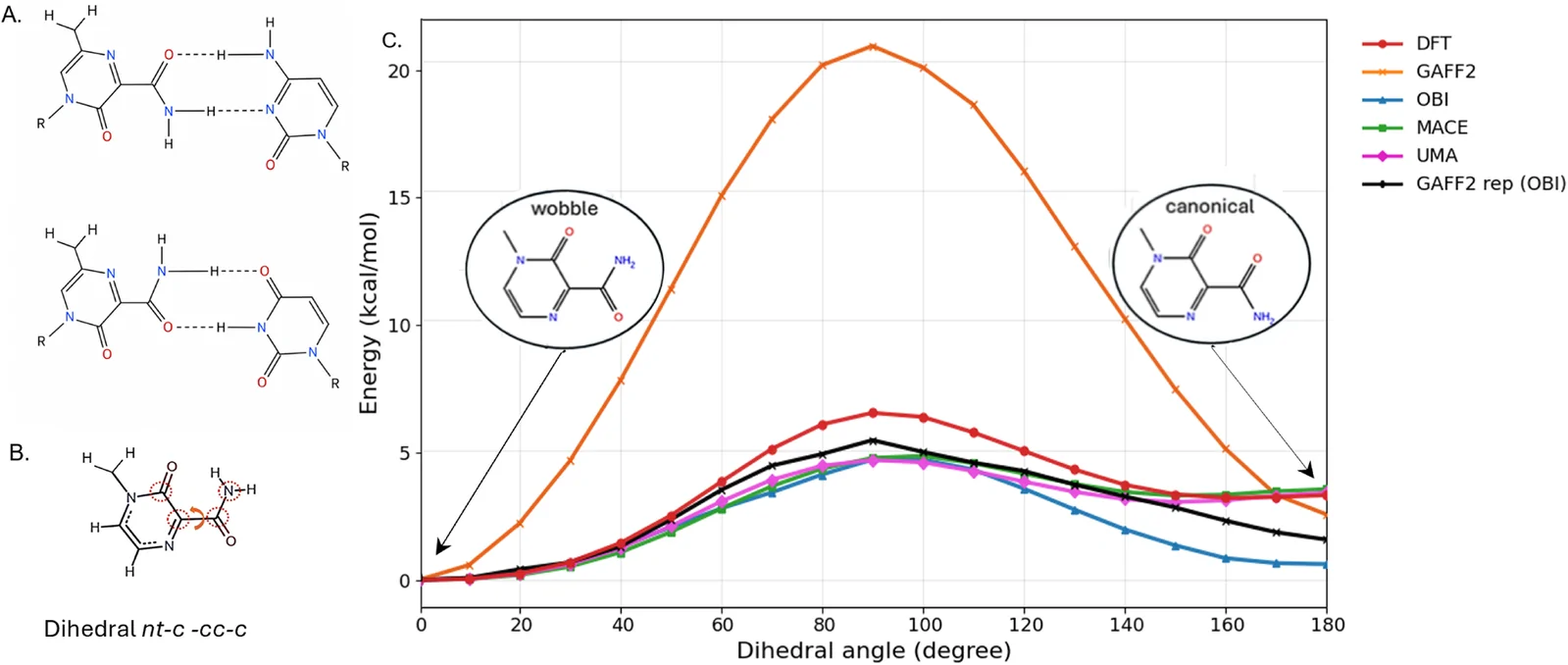
A) Molecule T1105, the defluorinated analog of favipiravir (T-705), base pairing with cytosine (left) and uracil (right).^46^ B) The four atoms highlighted with red dotted circles define the force field torsional term that was reparameterized. C) Comparison of GAFF2, MACE, OBIWAN, UMA and DFT profiles with the one obtained after the OBIWAN reparameterization (GAFF2 rep in the legend). Results from parameterization of GAFF2 based on MACE and UMA profiles are shown in Figure S1.

Using Torsionator in standard relaxed scan mode (*conf_analysis=false*), torsional energy profiles were generated using MACE, OBIWAN and UMA, all of which favorably compare to the dihedral scan performed at the DFT wB97X 6-31G­(d) level of theory as implemented in ORCA 6.0 (Figure 2C). Notably, the NNP considerably reduced the execution time of the torsional scan compared to DFT from ∼1 h to just a few minutes (Table S1). These profiles were used to reparameterize the single torsional term in GAFF2 (*m* = *0*) corresponding to the atoms highlighted in Figure 2B. The fitted parameters are reported in Table S1. Used to replace the original GAFF2 values, the torsional profiles align more closely with the DFT reference, correcting the overestimation of barrier observed with GAFF2, thus ensuring more realistic modeling of conformational transitions.

### Torsional Scan and Reparameterization of a Dihedral with Steric Hindrance

In the case of a molecule with multiple rotatable bonds, such as the drug emivirine, which features a central aromatic scaffold decorated with diverse functional groups, scanning a given dihedral angle may result in steric clashes between substituents. These clashes can result in either unrealistic energy spikes due to atomic overlaps that cannot be relaxed or discontinuities in the energy profile when coupled degrees of freedom abruptly change in response to the overlap. Both occurrences must be avoided to provide smooth torsional energy profiles for fitting. Torsionator allows addressing this issue by generating multiple conformers *(conf_analysis=true*), which are handled either via the Best Conformer Scan (*BCS=true)* or the Multi Conformer Scan (*MCS=true)*. We exemplify both approaches using emivirine as a test case.

Starting from the input experimental geometry of emivirine (PDB ID: 1RT1), Torsionator generated an ensemble of 40 initial conformers, which were filtered by RMSD, yielding 26 distinct conformers (including the experimental geometry). In BCS mode, a conformer is selected based on minimal steric interference, using Torsionator’s min–max Lennard-Jones criterion (see Methods section). This conformer is then used to scan the selected dihedral. This approach improves on performing the scan from a random structure (e.g., the crystallographic conformation), yet it may not be sufficient to resolve discontinuities in the torsional energy profile (Figure 3). In MCS mode, instead, each conformer is used to scan the selected dihedral, and the lowest pointwise energy values (with the corresponding geometries) are retained. To illustrate this behavior explicitly, we additionally plot the individual per-conformer energy profiles alongside the final MCS-derived profile for a representative dihedral (Figure S8). This procedure yields smooth torsional profiles suitable for parameterization; however, it is noteworthy that the scan trajectory does not form a continuous path, as it merges different conformers. Indeed, a smooth profile and a smooth scan trajectory may not be achievable simultaneously when scanning a single dihedral in a molecule with multiple rotatable bonds.

**Figure 3:**
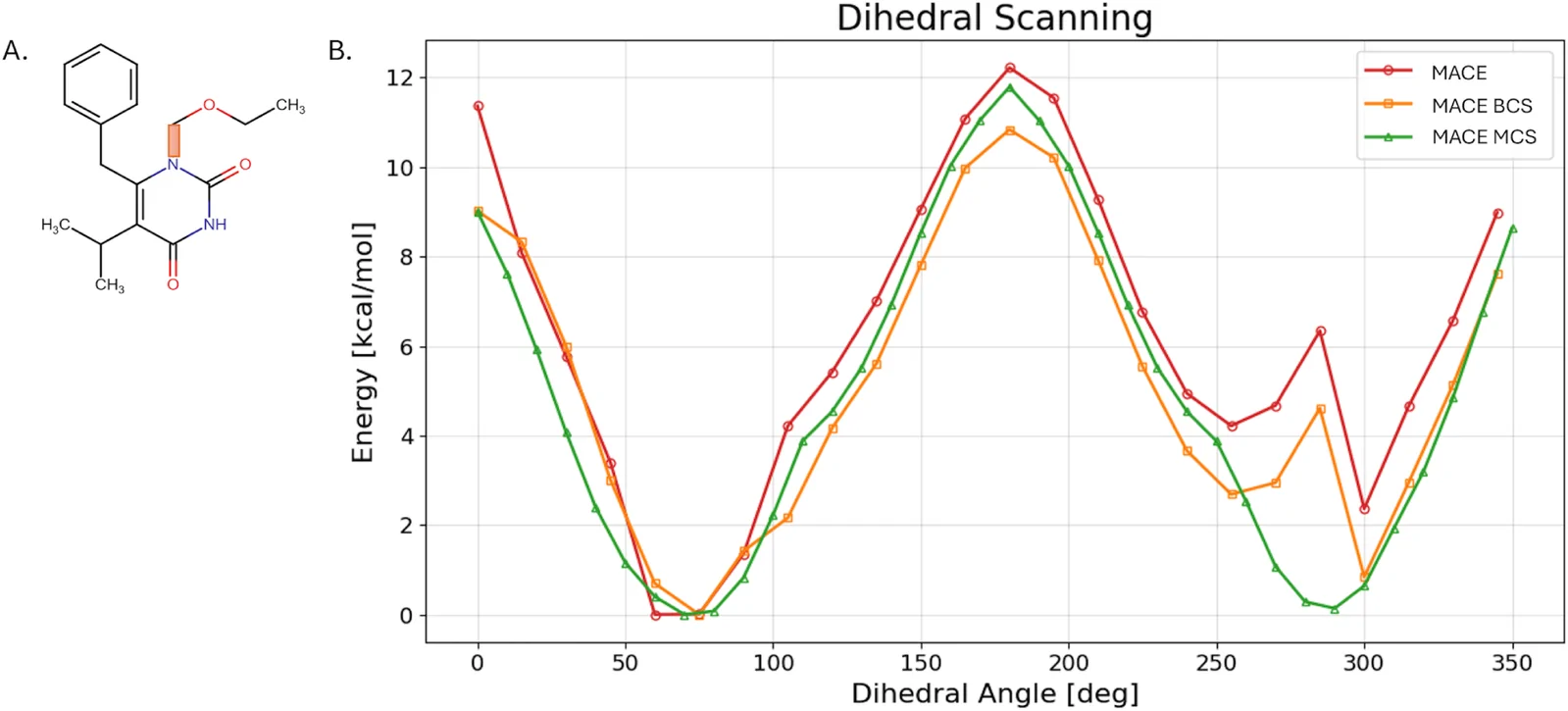
A) Chemical structure of emivirine, highlighting the rotatable bond investigated. B) MACE torsional scans performed in BCS and MCS modes compared to the scan performed from the crystallographic structure.

### Large Scale Validation

We tested the MCS procedure on the 500 drug-like fragments of the TorsionNet500 ^37^ database, which contains DFT torsion profiles computed at the B3LYP/6-31G** level. For this test we used MACE, whose accuracy with respect to the TorsionNet500 has already been established.^12^

We applied the MCS pipeline and adopted default settings (*RMSD=0.5 Å, n_confs=20*, *m=6*, *double_rotation=true*). A scan step of 15° was used to facilitate comparison with the TorsionNet500 reference data, whereas a scan step of 10° was used for parameter fitting. Our implementation proved to be robust, with torsional profiles close to the reference TorsionNet data for most molecules in the set. Geometries from the MCS procedure were also in line with the DFT ones, which were obtained using a similar approach. The RMSD between corresponding geometries is usually below 0.5 Å, showing a right-skewed distribution, with median of 0.24 Å and mean of 0.39 Å. Furthermore, smooth profiles suitable for fitting were obtained for most molecules in the set. Interestingly, the reparameterized GAFF2 yields lower RMSE (with respect to DFT) relative to GAFF2 in 83% of the torsional scans (Figure 4). We found the force constants that most improve are those of periodicity 2. The scans for which an improvement was not observed correspond to those fragments for which MACE itself deviates from the DFT reference.

**Figure 4:**
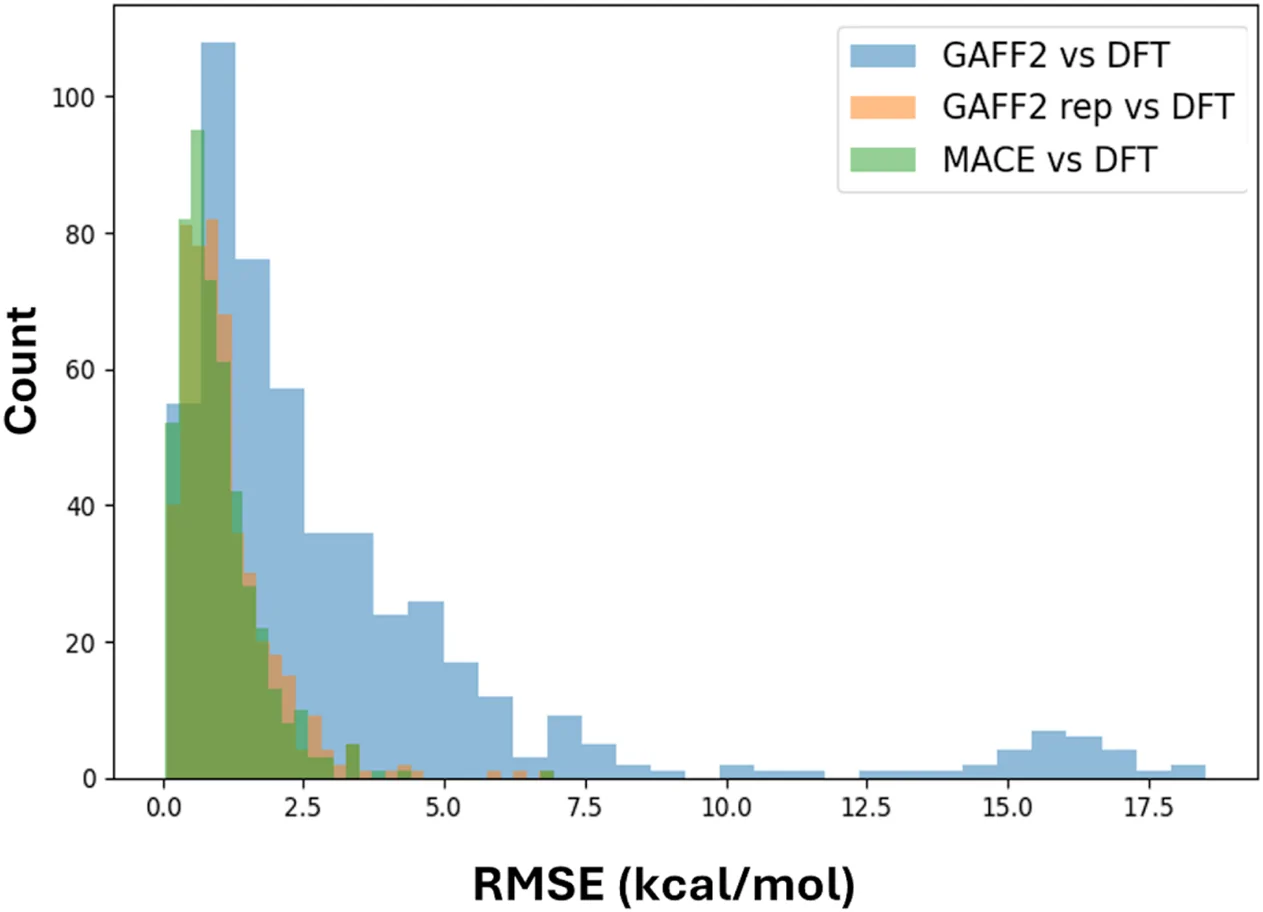
Histograms of the RMSE distribution of the original GAFF2, the reparameterized GAFF2 and MACE profiles with respect to the DFT reference data. The RMSE was computed for each torsional scan in the TorsionNet500 database.

### Conformational Analysis

Conformational diversity is a defining feature of small, flexible molecules and plays a decisive role in their physicochemical properties. This diversity is modulated by the environment and thus contributes, e.g., to binding affinities. For example, molecules often exist in solution as ensembles of conformers separated by small energy barriers, while only one conformer may be accessible within a protein target-binding pocket. For this reason, Torsionator integrates conformer generation as a core component of its pipeline. We exemplify the workflow using again the drug emivirine (Figure 5A), a known non-nucleoside inhibitor of HIV reverse transcriptase.^47^

**Figure 5:**
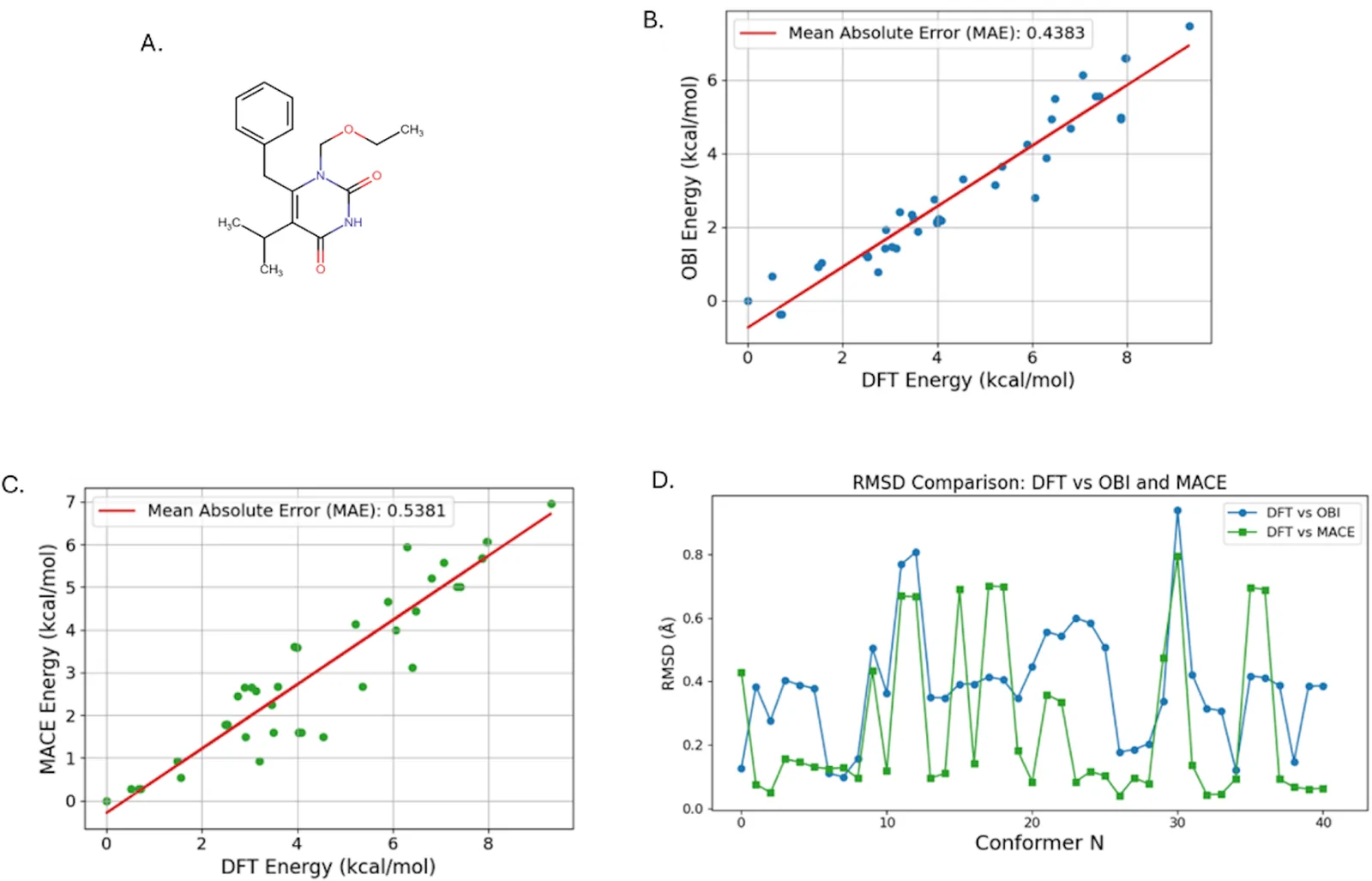
A) Chemical structures of of emivirine. B) Correlation between OBIWAN and DFT conformational energies. C) Correlation between MACE and DFT conformational energies. D) RMSD between DFT optimized geometries and the corresponding ones optimized with MACE and OBIWAN.

Starting with the input experimental geometry (PDB ID: 1RT1), Torsionator generated an ensemble of 40 conformers. All conformers, along with the crystallographic structure, were subsequently optimized using both OBIWAN and MACE. For reference, the set of conformers was also optimized at the DFT wB97X 6-31G­(d). Figure 5 shows the correlation between relative conformer energies predicted by OBIWAN and MACE with respect to those from DFT. Both NNPs capture the broad energetic ordering of the conformers, with a mean absolute error of ∼0.5 kcal/mol relative to DFT. Furthermore, the RMSD between DFT and NNPs-optimized geometries is <1 Å (average RMSD is ∼0.39 Å for OBIWAN and ∼0.24 Å for MACE).

Regarding the energy ranking, DFT and MACE identified the same conformer as the global minimum, which differs from the crystal-bound structure, whereas OBIWAN converged on a different conformer. However, the energy difference between the pair of conformers was 0.38 kcal/mol. These results confirm that Torsionator can conveniently be used to navigate the conformational landscape of small molecules.

## Conclusions

Here, we present Torsionator, a software tool that enables rapid dihedral scanning and scalable exploration of conformational space without compromising chemical accuracy, using neural network potentials (NNPs) and currently supporting OBIWAN, MACE, and UMA. By integrating such NNPs, Torsionator allows conformer analysis, clash-free and multi-conformer scanning, and automated reparameterization into a single workflow. The case studies presented here demonstrate that NNP-derived torsional profiles, conveniently obtained with Torsionator, can be fitted to correct deficiencies of generic force fields, while requiring only a fraction of the DFT computational cost. This also rendered the parameterization task more energetically sustainable in computational studies. Given these results, we plan to develop Torsionator further and support additional NNPs in the future.

## Supplementary Material



## Data Availability

The software Torsionator and the code can be accessed at: https://github.com/giobros/torsionator.git. The repository also contains an Apptainer recipe and pre-built image for ease of installation. The frcmod and mol2 files obtained after the reparameterization of molecule T1105, as well as the TorsionNet500 profiles obtained with Torsionator, can be found in https://github.com/giobros/torsionator/releases/tag/Torsionator.

## Notes

Technical details on the Torsionator tool, supported modes, additional profiles, and comparative analyses across the different operational modes (PDF)
