# Supplementary material for: Torsionator: Dihedral Parameterization and Conformer Search Made Easy
Source: J Chem Theory Comput. 2026 Sep 9;22(19):10419–27. doi: 10.1021/acs.jctc.6c01006 (PMC13671165; doi:10.1021/acs.jctc.6c01006)
Supplement: Supplementary file 1 [file ct6c01006_si_001.pdf]

Torsionator: dihedral parameterization and conformers search made easy

Giorgia Brosio[1], Alice Triveri[1], Pietro Vidossich[1], Sergio Decherchi[1], Marco De Vivo*[1]*

1. Laboratory of Molecular Modelling & Drug Discovery, Fondazione Istituto Italiano di Tecnologia, Via Morego 30, 16163, Genoa, Italy

Supporting Information

Contents:

1. **Geometry optimizations and torsional scans**
2. **Conformer generation, clash detection and best conformer selection**
3. **Examples**
4. **Large scale testing on TorsionNet500**

1. Geometry optimizations and torsional scans

Geometry optimizations and relaxed torsional scans were performed using the Atomic Simulation Environment (ASE)[1] , which accesses NNPs forces from the respective interfaces. For the initial structural relaxation from the input geometry, we found convenient to start with some gradient-descent steps (100 steps) and next switch to the BFGS optimizer (max steps=1000). For torsional relaxed scans, only BFGS was used. A tolerance of 1e-4 eV/Å on the maximum force on any atom was used as a convergence criterion. Torsional relaxed scan used the FixInternals functionality from ASE. The calculations are executed in a vacuum (gas-phase) consistently with standard practice in the field[2,3]. Solvent effects that may alter the relative energies of torsional profile, were not considered in this work.

The number of scan points is controlled by the *scan_step* keyword, resulting in 360°/*scan_step* constrained optimizations. The geometries.xyz file from the ASE/NNP optimization step, as well as the sqm.pdb file from the antechamber/AM1 optimization contain the atomic connectivity.

2. Conformer generation, clash detection and best conformer selection

A conformer ensemble is generated using the Experimental Torsion Knowledge Distance Geometry (ETKDG) algorithm as implemented in RDKit[4]. ETKDG extends the classical distance geometry approach by incorporating experimentally derived torsional preferences from the Cambridge Structural Database (CSD) together with basic chemical knowledge, producing chemically realistic three-dimensional conformations. The algorithm constructs a distance bounds matrix from the molecular topology, applies triangle-inequality smoothing, generates an initial three-dimensional embedding by metric distance geometry, and subsequently refines the structure using knowledge-based torsional and geometric constraints. By default 20 conformers are generated. Conformers are initially minimized using MMFF94s and pruned based on an RMSD cutoff (default: 0.5 Å). Each conformer in the resulting set is then minimized with the NNP of choice.

For each conformer, a preliminary exploratory scan of the selected dihedral is performed in 10° increments to evaluate steric clashes. Interatomic distances are computed and if any pairwise distance falls below the sum of the corresponding van der Waals radii minus a buffer of 0.3 Å the conformer is flagged. Steric interactions are estimated between the rotating fragments using a Lennard–Jones (LJ) potential, excluding 1–2, 1–3, and 1–4 bonded pairs. The rotating fragments associated with a dihedral angle a–b–c–d are defined by a two-front breadth-first search procedure on the molecular graph (in case the input pdb file lacks the CONNECT records, the molecular connectivity is inferred with OpenBabel[5]). This results in a set comprising atoms on the *-b side of the dihedral and a group of atoms on the c-* side. The maximum LJ interaction energy observed over the full rotational scan is recorded for each conformer as a measure of steric strain. In addition, a mass-weighted radius of gyration (Rg) is computed for each conformer, and the average conformer radius of gyration ⟨Rg⟩ is tracked along the scan. The maximum ⟨Rg⟩ value is retained as a complementary descriptor of molecular extension and compactness.

The best conformer is the lowest-energy conformer that displays clash-free rotation. If no conformer is clash-free, the conformer with minimal LJ energy is selected.

3. Examples

Favipiravir de-fluoro analog T1105. The rotatable bond of favipiravir de-fluoro analog T1105 was scanned in 10° increments. Profiles obtained with the NNPs MACE[6,7], OBIWAN[8] and UMA[9], the GAFF2 force field and the reparametrized variants are compared to the DFT reference profiles in Figure S1, using a fit restricted to the GAFF2 original n=2 term of the Fourier series (setting m=0 in the software), and m=9 (n=1,2,3,4,5,6,7,8,9). Force field parameters of the nt -c-cc -c term derived from fitting the NNPs torsional scans, are consistent with those derived from DFT data (Table S1).

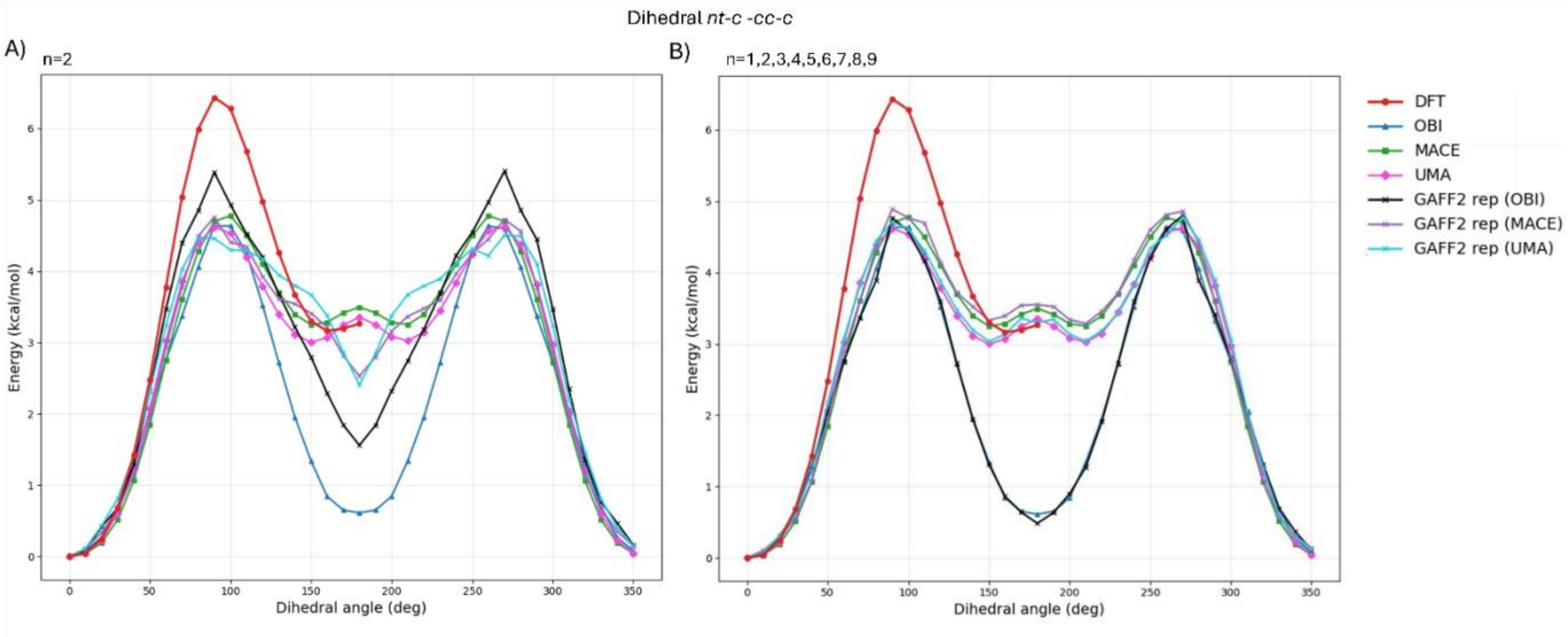

Figure S1: Comparison of MACE, OBIWAN and UMA torsional profiles with the ones obtained after the re-parametrization (GAFF2 rep in the legend) of the *nt -c-cc -c* term for molecule favipiravir de-fluoro analog T1105 using m=0 (n=2) (A) and m=9 (n=1,2,3,4,5,6,7,8,9) (B) The DFT profile is also included for comparison.

Method	K [kcal/mol]	γ [degrees]	n	Error [kcal/mol]	Time (scan 0 - 180)
**DFT**	-4.351	180.0	2.0	-	~ 1h, 9m
**GAFF2**	11.500	180.0	2.0	15.851	< 1s
**MACE**	-5.031	180.0	2.0	0.680	~ 1 m 30 s
**OBIWAN**	-4.428	180.0	2.0	0.077	~ 3 m 15 s
**UMA**	-4.754	180.0	2.0	0.403	~ 2 m 40 s

Table S1: Comparison between the parameters of the *nt -c-cc -c* term obtained using DFT, GAFF2, MACE, OBIWAN and UMA using GAFF2 original n=2 term. Computational times are also reported. NNPs were run on an AMD Ryzen Threadripper 2-Cores workstation with a NVIDIA RTX A4000. DFT calculations were run on 4 cores of an AMD EPIC node of the IIT Franklin cluster.

Imatinib. This kinase inhibitor displays multiple rotatable bonds. Torsional profiles for five dihedrals were computed with MACE, OBIWAN and DFT using the relaxed scan mode. The profile obtained with relaxed scan methodology shows spikes, that are present and coherent across both the DFT reference and NNPs predictions. To obtain a profile without jumps we performed a scan using MCS=true for the rotatable bonds with discontinuities (purple and red ones in Figures S2, S3). Result profiles calculated with both MACE and DFT are shown in Figure S3. Profiles computed in MCS mode are compared to single point DFT energy calculations on MCS geometries using the wB97X-D3 functional and the def2-TZVP basis-set.

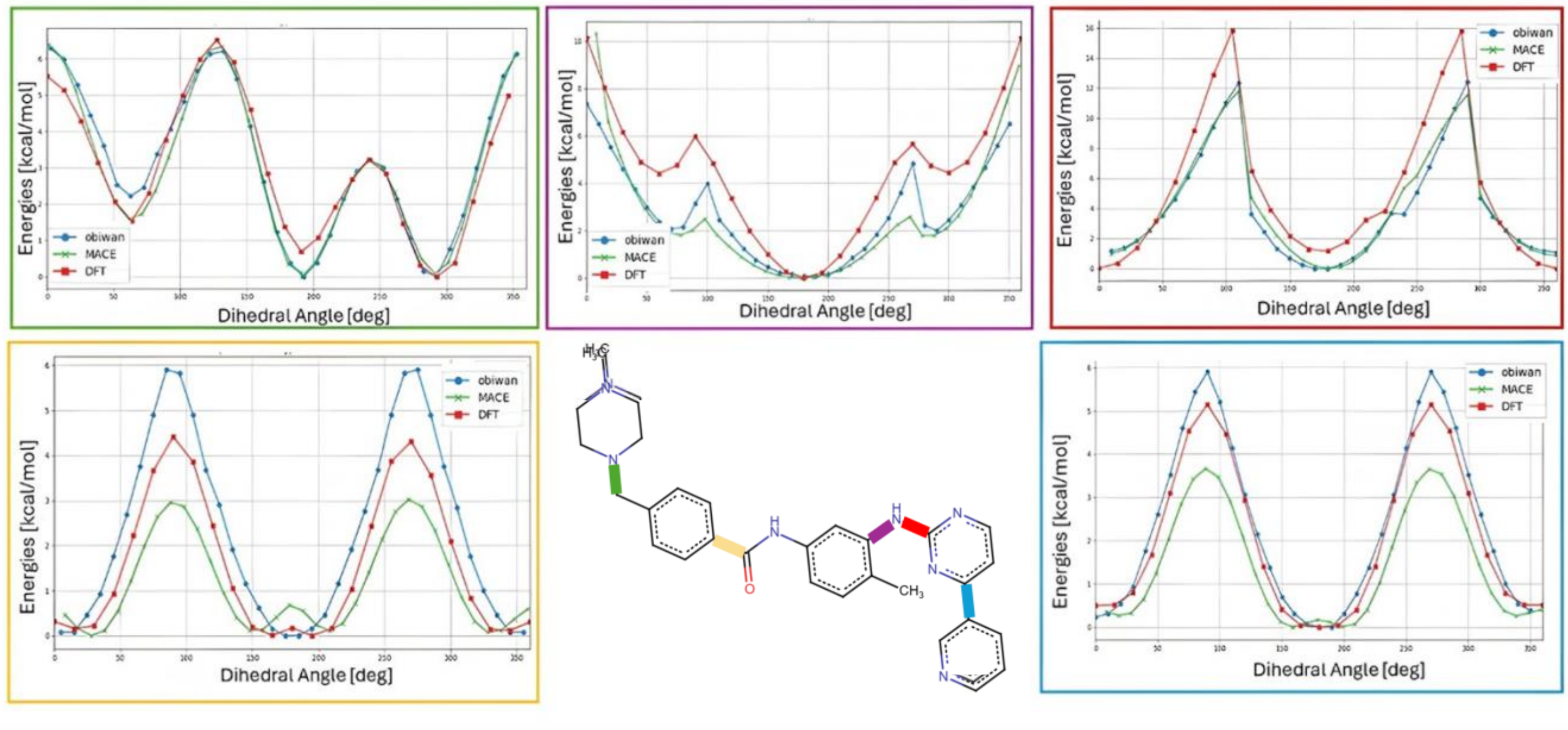

Figure S2: Comparison of MACE, OBIWAN and DFT energy profiles obtained with the relaxed scan procedure for five dihedrals of the drug imatinib. The rotatable bonds investigated are color-coded in the molecular structure; their corresponding energy profiles are outlined in matching colours for easy visual correspondence.

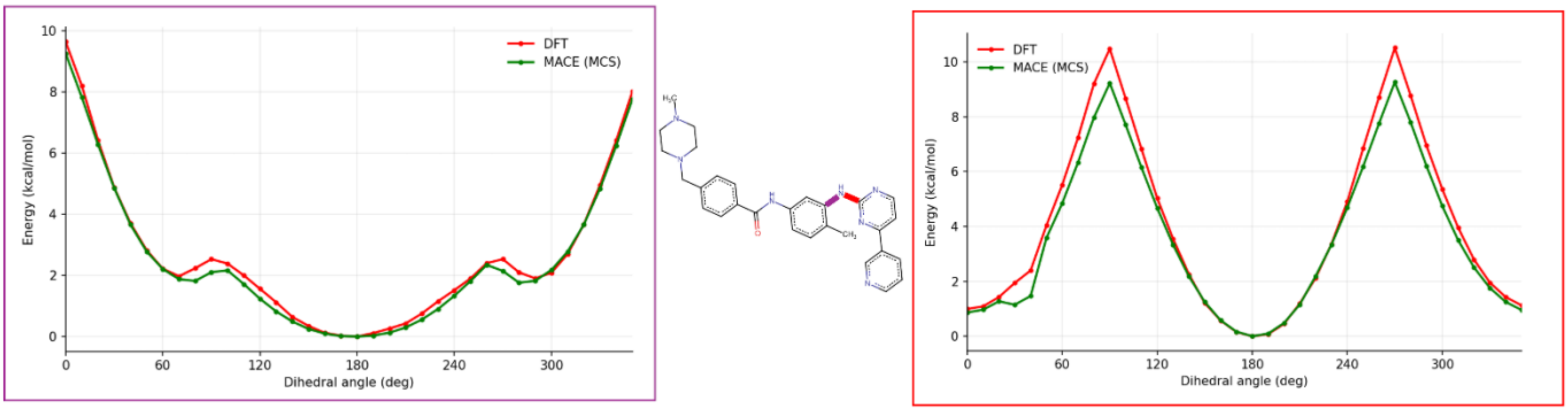

Figure S3: MACE and DFT energy profiles computed with the MCS protocol for the two dihedrals highlighted of the drug imatinib, to be compared with Figure S2, where normal Relaxed Scan produced spikes.

Charged molecules. The small model of Universal Models for Atoms (UMA) is supported to extend the applicability domain of the framework to systems with non-zero net charge, as it was trained on the OMOL25 dataset, which includes charged species. The molecular charge is passed to the workflow via the keyword *net_charge*. We exemplify results on diphosphate (charge -2) and carbamylcholine (charge +1). Profiles computed in MCS mode are compared to single point DFT energy calculations on MCS geometries using the B3LYP functional and the 6-31G* basis-set

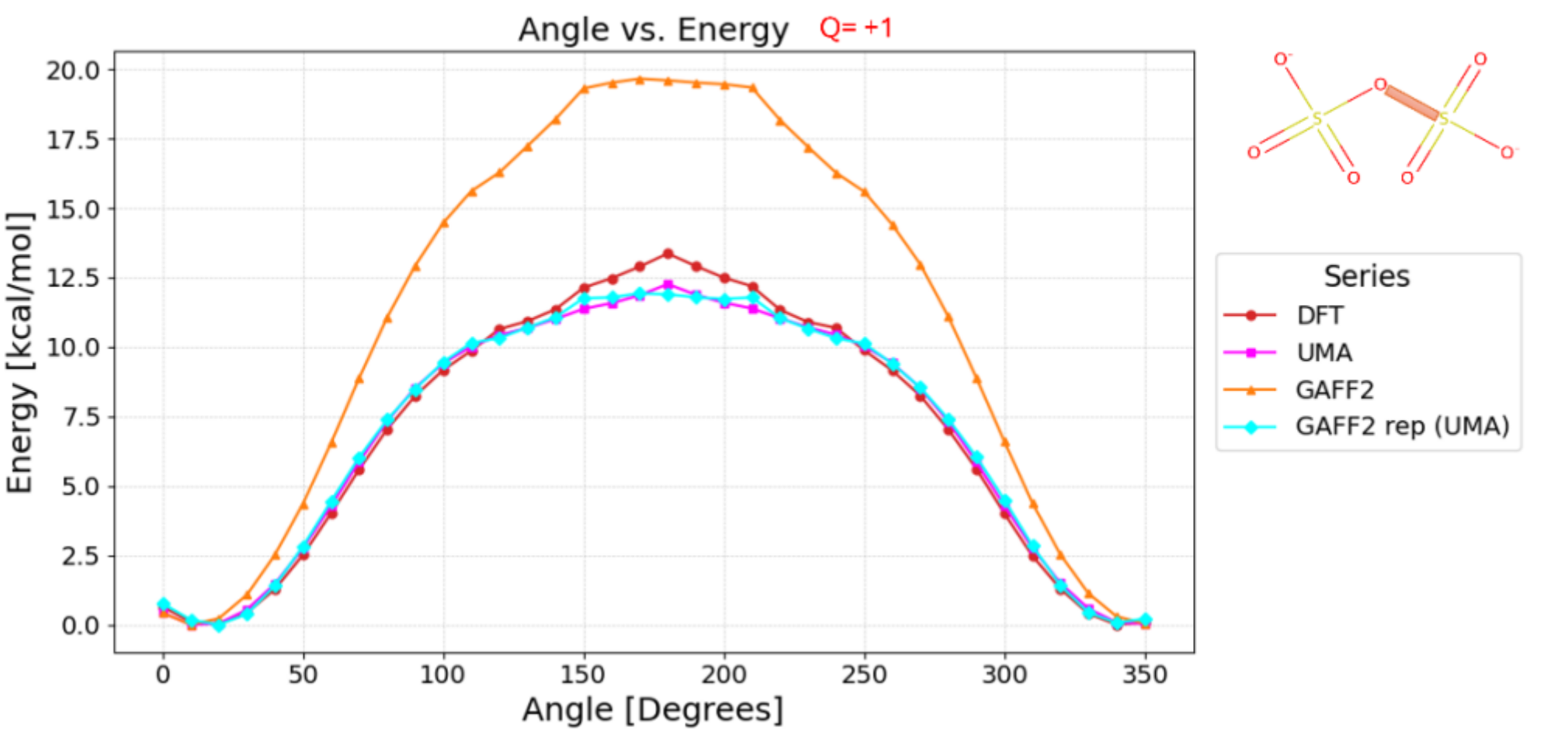

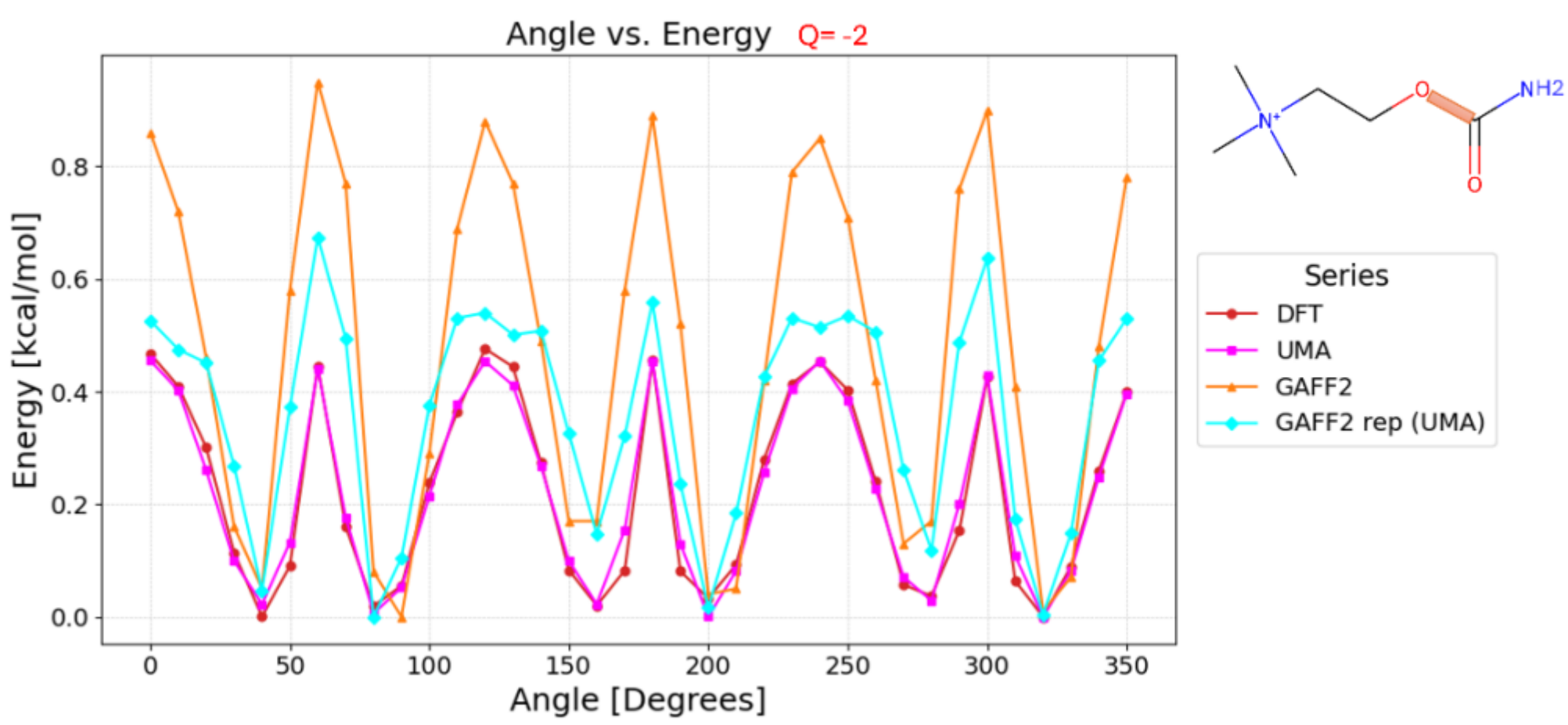

Figure S4: UMA torsional scans compared with DFT (B3LYP/6-31G*) single point energy evaluations. The ratable bond is highlighted in orange in the molecular diagram. Torsionator was run with *MCS=true, step_size*=10, *m=6* and setting the molecular charge with keyword *net_charge*.

4. Large scale testing on TorsionNet500.

We tested the MCS procedure on the full set of 500 drug-like small molecules included in the TorsionNet500[10] benchmark, which includes DFT geometries and energies of torsional scans computed at the DFT level. All molecules were successfully processed by Torsionator (runs were performed using the MACE potential only). For most molecules, the RMSD between geometries from Torsionator falls below 0.5 Å from those distributed with TorsionNet500 (Figure S5). Furthermore, the reparametrized force field displays in general better agreement with the DFT data than the original GAFF2. However, there are instances for which the MACE potential did not result in accurate profiles (compared to the DFT data), resulting in reparametrized torsional terms that perform worse than the original GAFF2 (Figure S6).

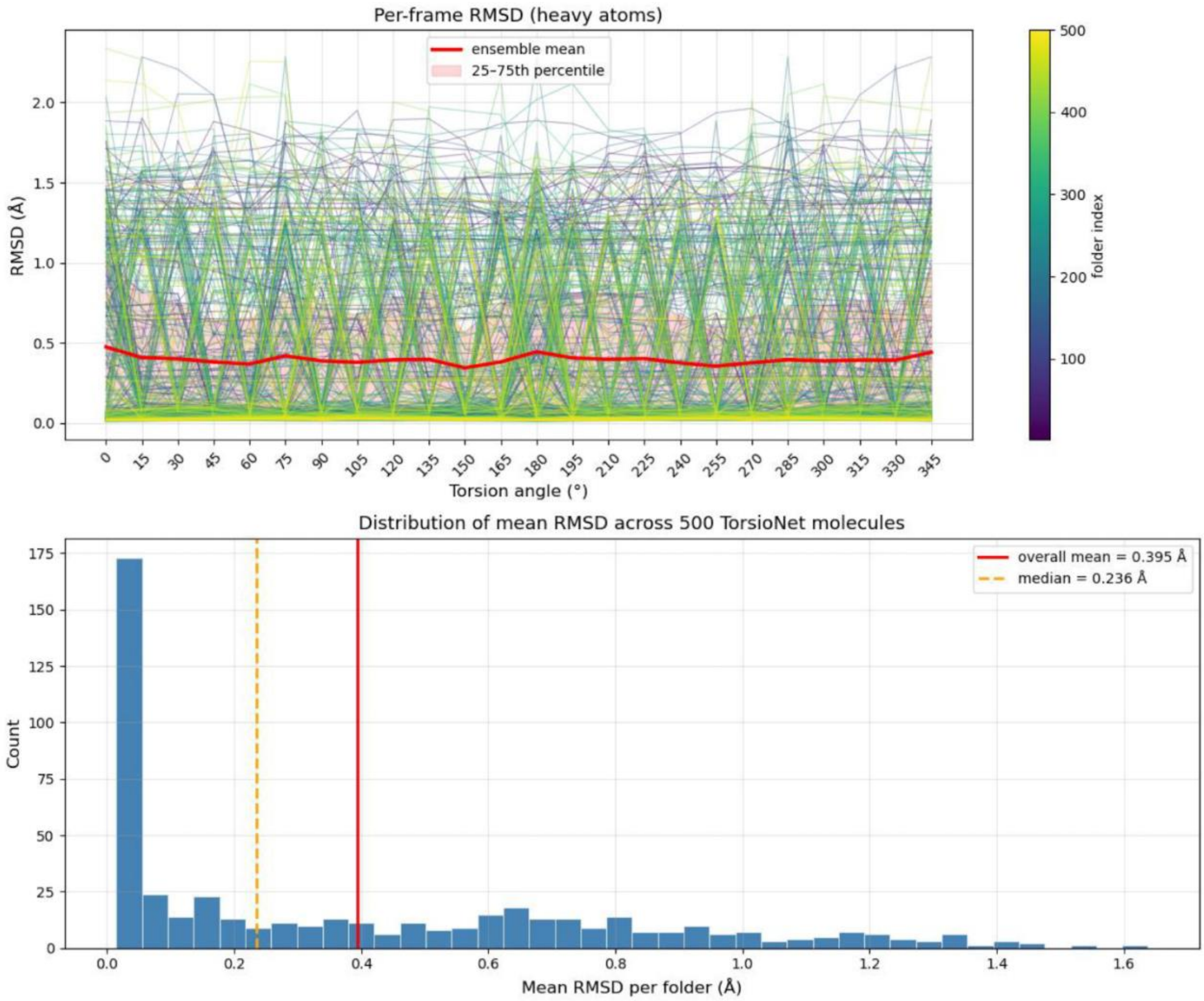

Figure S5: Assessment of Torsionator (based on MACE) geometries against DFT reference structures for the TorsionNet500 data set. The RMSD between corresponding geometries was computed after optimal alignment of heavy atoms. *(Top)* Per-frame RMSD as a function of torsion angle. The red line denotes the ensemble mean across all molecules; the shaded band covers the 25th–75th percentile range. The mean RMSD is

approximately constant across all angles (~0.4 Å), indicating no systematic angular dependence in the MACE predictions. *(Bottom)* Distribution of the per-molecule mean RMSD (averaged over the 24 scanned frames). The distribution is right-skewed, with a median of 0.24 Å and a mean of 0.39 Å.

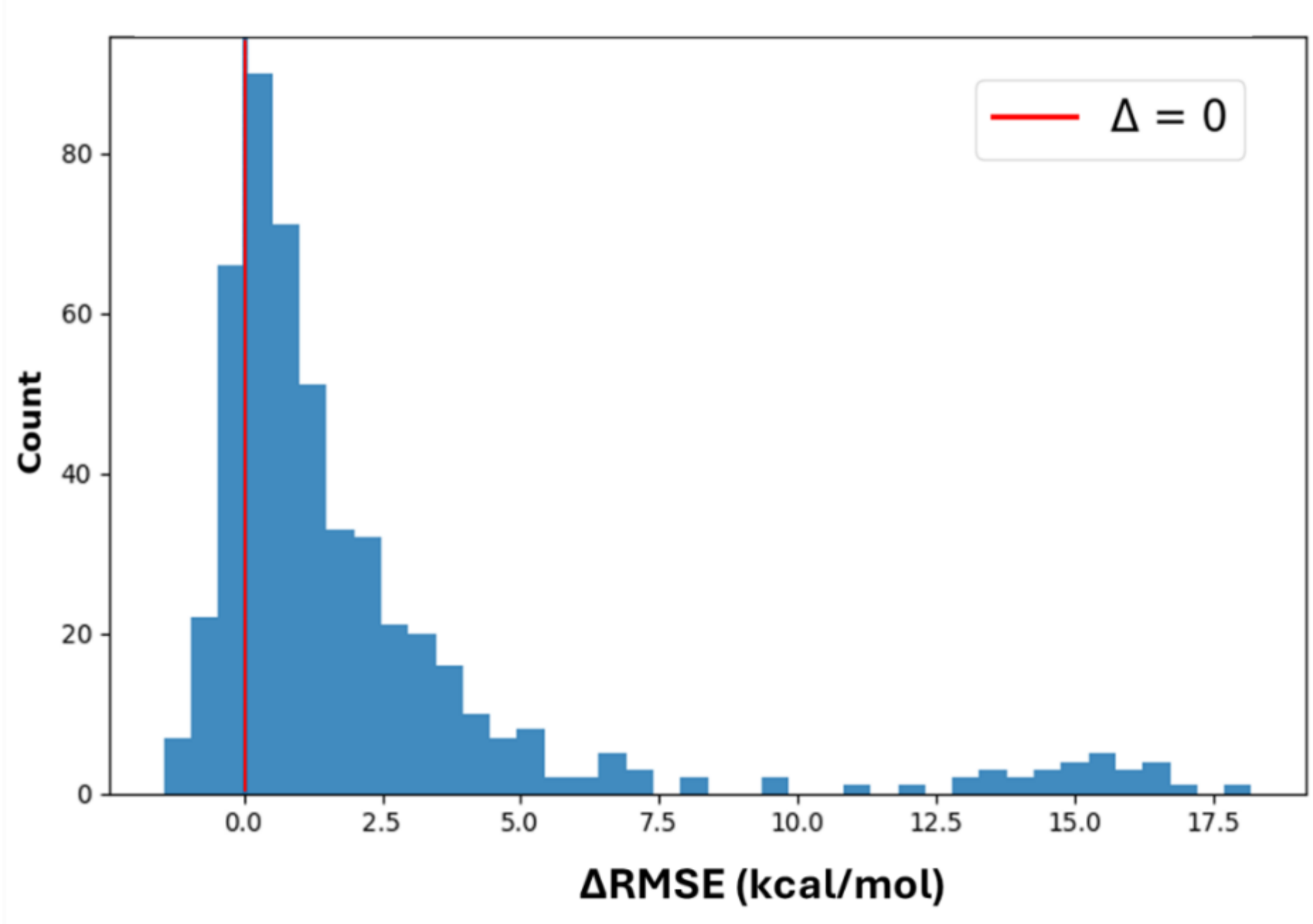

Figure S6: A) Histograms of the distribution of the ΔRMSE = RMSE(GAFF2 vs DFT) – RMSE(GAFF2 rep vs DFT) obtained by applying Torsionator to the TorsionNet500 data set. Positive values indicate a better result for GAFF2 rep.

MCS mode. As noted in the main text, a major issue in performing a torsional scan in a molecule with multiple rotatable bonds is that discontinuities may appear in the computed energy profile. The MCS procedure addresses this issue, in general providing smooth profiles more suitable for parameter fitting than single conformer scans (including the BCS mode), as shown in Figure S7.

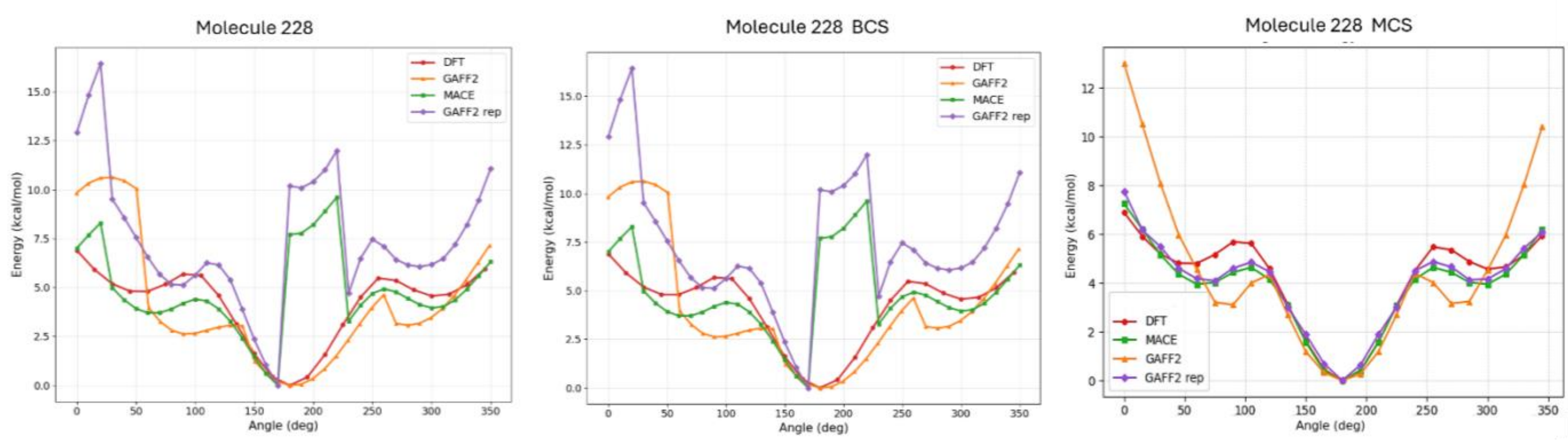

Figure S7: Examples of the scanning of one molecule (top and bottom rows) using all three Torsionator modes : left) scanning the input structure, middle) the best conformer one, and right) the MCS multi-conformer approach. Chemical structure of the molecule in Figure S11.

To further illustrate the MCS method in detail, we plot the energy of each conformer in the ensemble as a function of the torsion angle, using molecule 27 from TorsionNet500 as a representative example shown in Figure S8

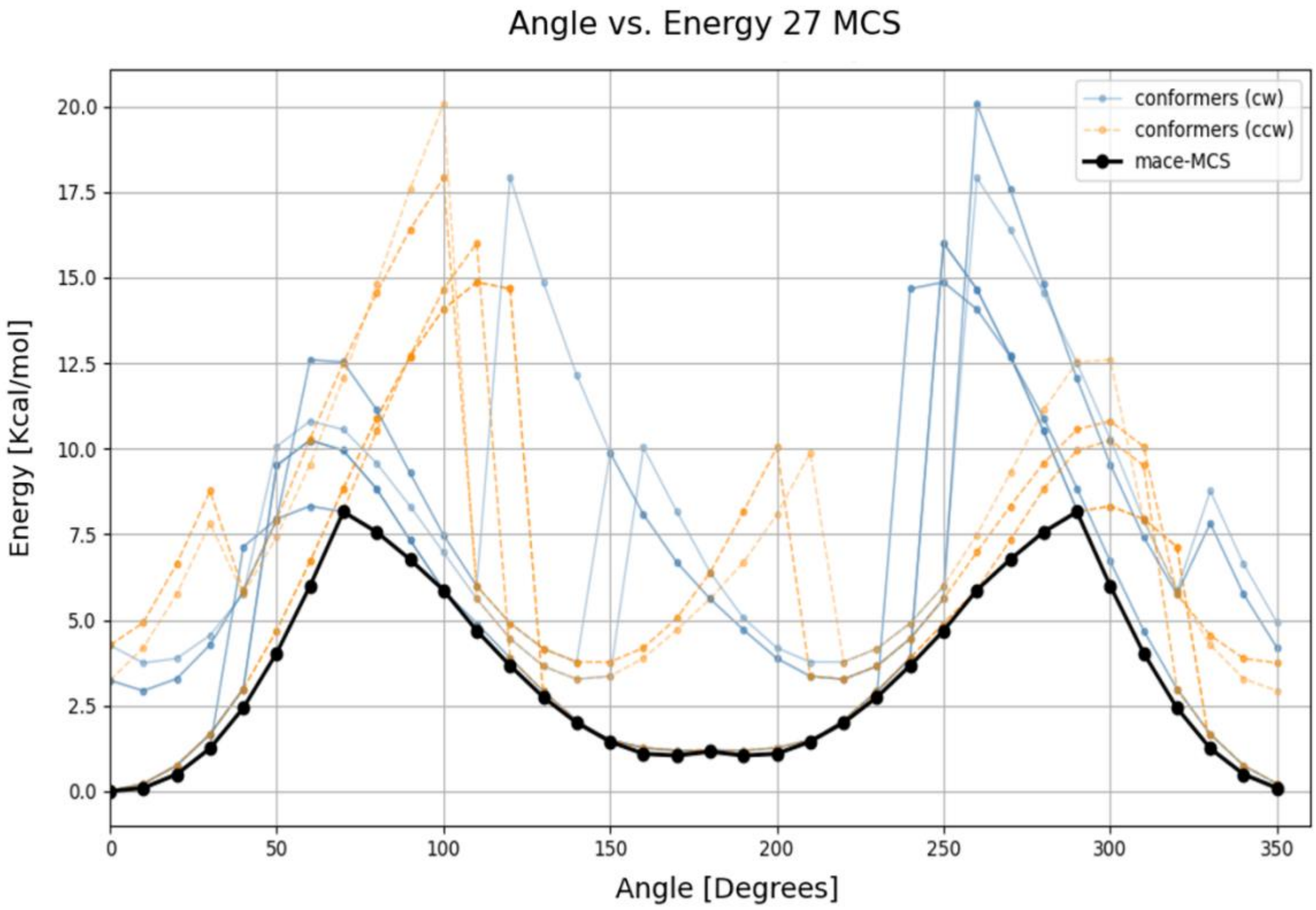

Figure S8: Energy profiles of the eight conformers (light blue) remaining after screening as a function of the dihedral angle, together with the resulting MCS energy profile (black) obtained with the MACE potential. At each angle, the MCS curve corresponds to the lowest-energy conformer in the ensemble, tracing the effective torsional barrier. Chemical structure of the molecule in Figure S11.

The *double_rotation* option further addresses the effect of hysteresis. In Figure S9, we compare results from the MCS workflow performed with different settings of this variable. When *double_rotation=false* scans are performed either clockwise or counterclockwise. This may result in distinct energy profiles, exhibiting mirrored behavior along the angular coordinate. Consequently, the resulting torsional profile is asymmetric and may introduce artifacts in the subsequent parameterization. When setting *double_rotation=true* the scan is performed in both directions for each conformer, and the lowest-energy structures at each dihedral angle is retained. This approach effectively removes hysteretic effects, displaying the sought symmetry of the torsional energy profile. The resulting MACE-derived profile is smooth, demonstrating that the inclusion of bidirectional scanning is beneficial for obtaining physically consistent torsional profiles suitable for parameter fitting. It may also be observed that the MACE profile is in good agreement with the DFT data from TorsioNet.

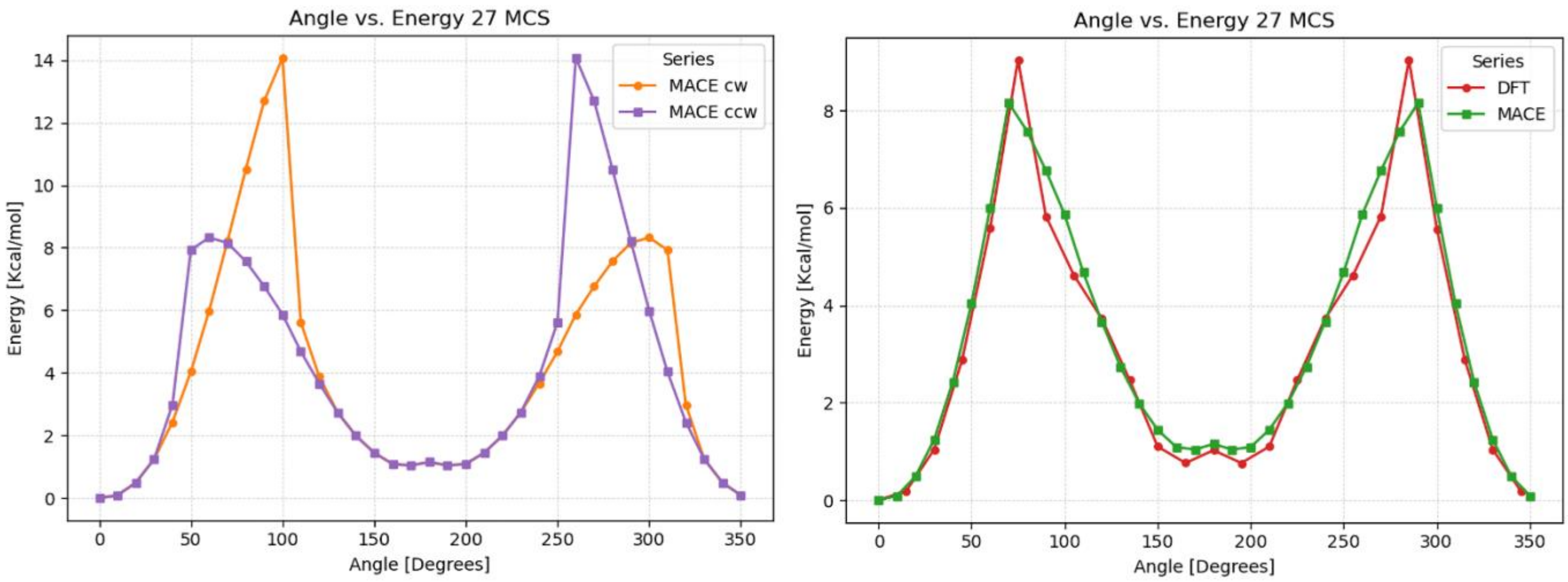

Figure S9. Left) Torsional scan obtained using Torsionator in MCS mode with *double_rotation=false*. Independent scans were performed in clockwise (cw) and counterclockwise (ccw) directions. Right) The scan obtained using *double_rotation=true* restores the expected symmetric profile. Chemical structure of the molecule in Figure S11.

Role of regularization during fitting. The effect of the *hrst* regularization parameter is shown in Figure S10, where the full family of GAFF2 reparametrized, torsional profiles generated by varying the *hrst* parameter is reported. Within this family, the profile highlighted in purple shows the closest agreement with the MACE reference, minimizing the deviation (RMSE) over the entire dihedral range, and was therefore selected as the final, optimal reparametrized model (GAFF2 rep profile). This further fine tuning allows to get an improved matching to the reference profile.

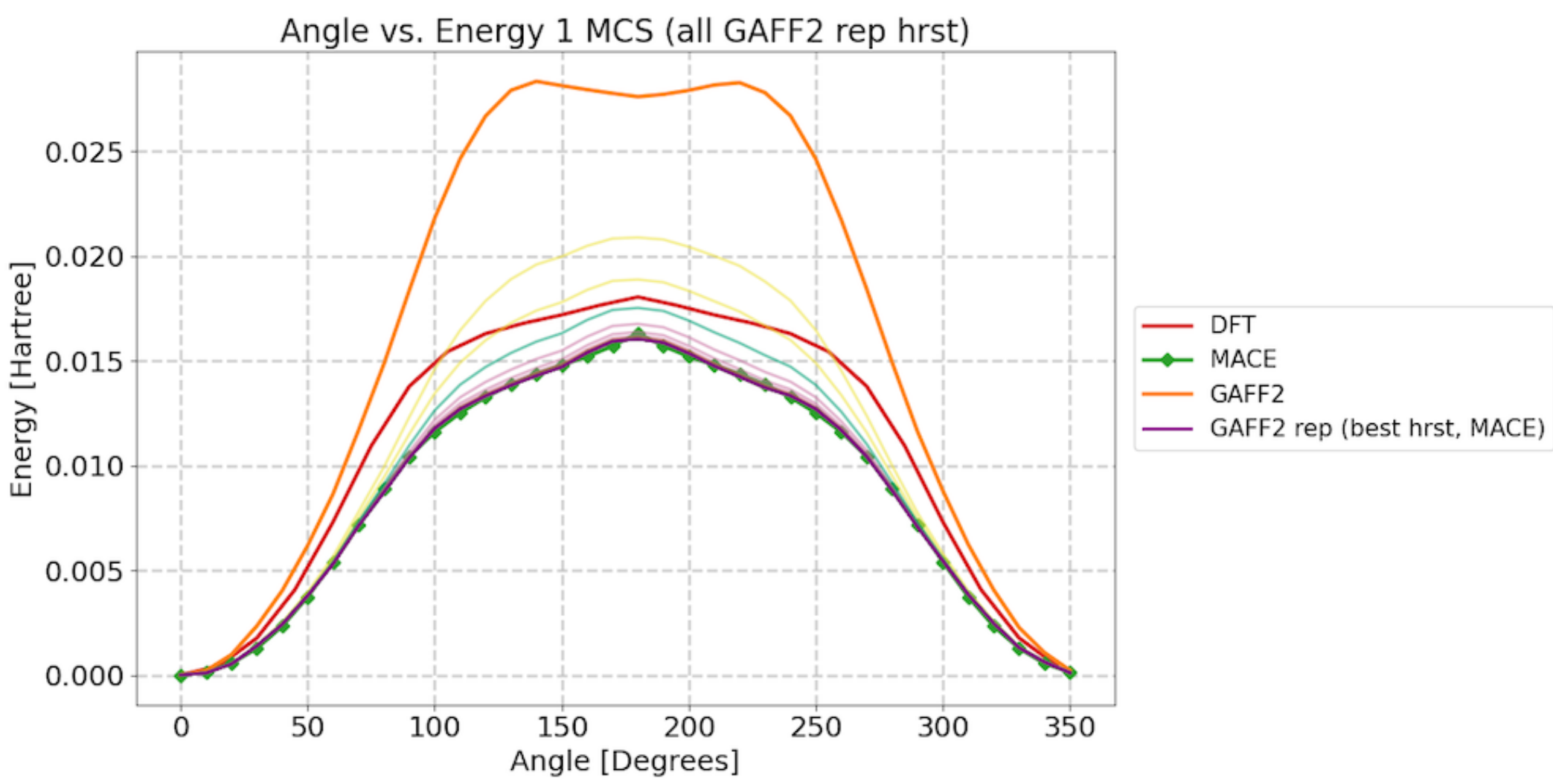

Figure S10: Family of GAFF2 rep reparametrized profiles obtained varying the *hrst* regularization parameter. The purple profile shows the smallest RMSE with respect to the MACE data. The chemical structure of the molecule is reported in Figure S11.

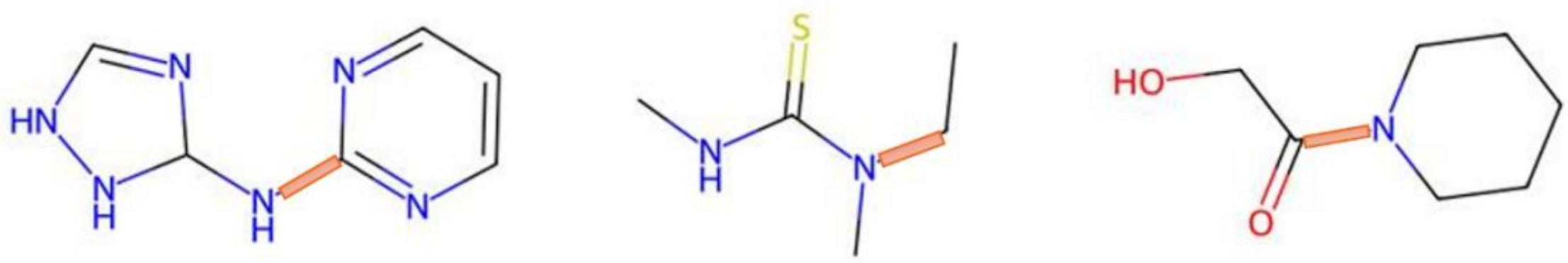

Molecule 1 Molecule 27 Molecule 228

Figure S11: Chemical structure of the three TorsioNet500 molecules used for the torsional scans reported in Figures S7-S9-S10. The scanned dihedral is highlighted.

Aromatic and Heteroaromatic connecting torsions. Torsions connecting aromatic and heteroaromatic rings are particularly challenging, as the rotational potential arises from a delicate balance between π-conjugation across the inter-ring bond, steric repulsion, and nonbonded interactions. Such torsions are ubiquitous in drug-like molecules and have long been recognized as difficult to describe accurately with transferable force fields such as GAFF/GAFF2. Figure S12 illustrates the fit for four molecules from TorsioNet500 which includes about 200 structures N-aryl or biaryl-like.

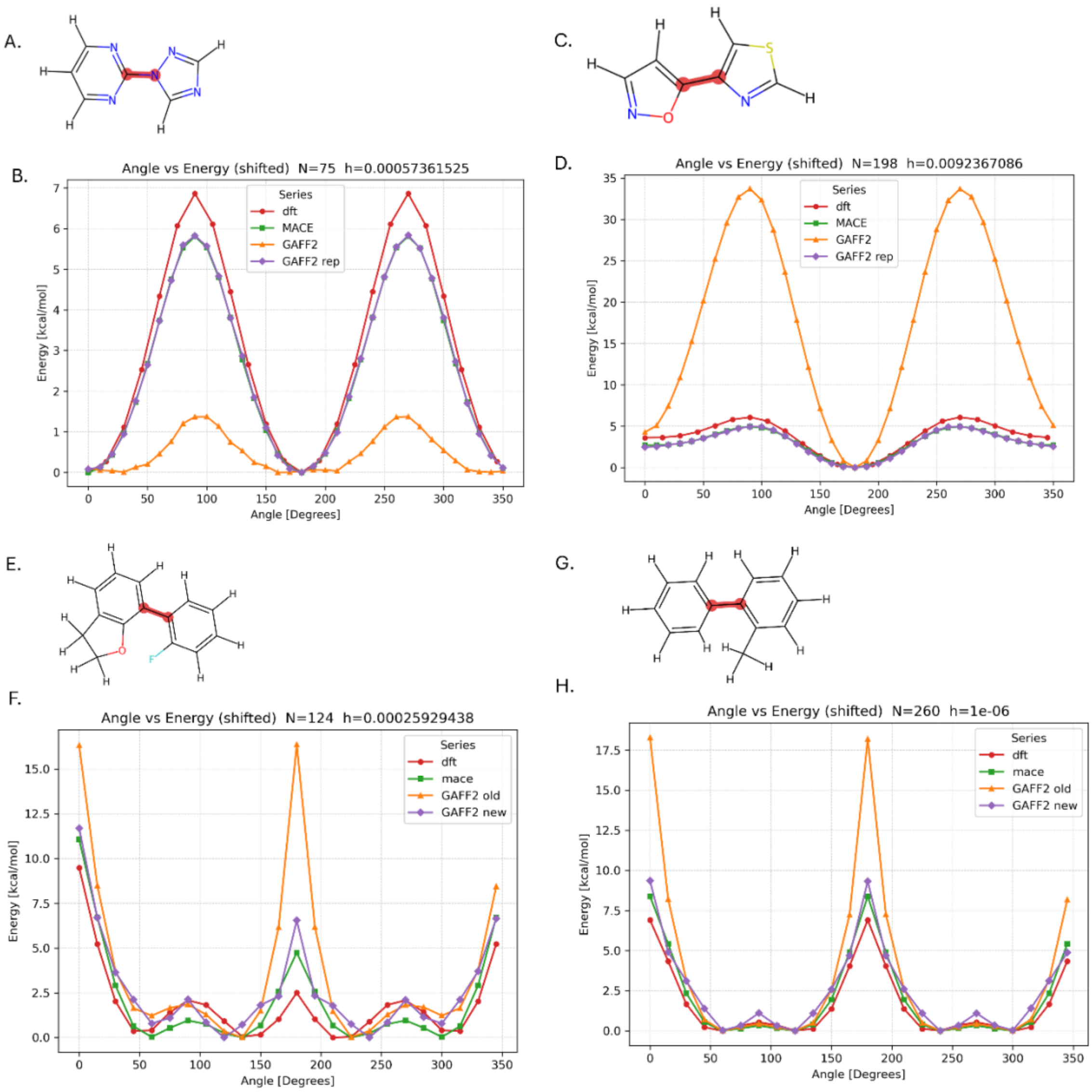

Figure S12: Four representative aromatic–aromatic torsions. In the first example (A–B), GAFF2 underestimates the rotational barrier (~1 kcal mol^{-1} versus ~7 kcal mol^{-1} from DFT), whereas both MACE and the GAFF2 rep model closely reproduce the DFT profile over the entire scan. In the second example (C–D), GAFF2 instead overestimates the barrier (~33 kcal mol^{-1} versus ~6 kcal mol^{-1} from DFT), while MACE and GAFF2 rep again remain in close agreement with the quantum-mechanical reference. The third (E–F) and fourth (G–H) examples show that the original GAFF2 parameters overestimate the barriers by several kcal mol^{-1} relative to DFT, while MACE and GAFF2 rep both track the DFT profile closely across the full scan.

Selection of the Fourier term set (*m* parameter)

m=0	n= GAFF2
m=1	n=1
m=2	n=1,2
m=3	n=1,2,3
m=4	n=1,2,3,4
m=6	n=1,2,3,4,6
m=9	n=1,2,3,4,5,6,7,8,9

Table S2: Sets of periodicity terms n included in the Fourier expansion as a function of the parameter *m* passed to Torsionator.

References

(1) Hjorth Larsen, A.; Jørgen Mortensen, J.; Blomqvist, J.; Castelli, I. E.; Christensen, R.; Dułak, M.; Friis, J.; Groves, M. N.; Hammer, B.; Hargus, C.; Hermes, E. D.; Jennings, P. C.; Bjerre Jensen, P.; Kermode, J.; Kitchin, J. R.; Leonhard Kolsbjerg, E.; Kubal, J.; Kaasbjerg, K.; Lysgaard, S.; Bergmann Maronsson, J.; Maxson, T.; Olsen, T.; Pastewka, L.; Peterson, A.; Rostgaard, C.; Schiøtz, J.; Schütt, O.; Strange, M.; Thygesen, K. S.; Vegge, T.; Vilhelmsen, L.; Walter, M.; Zeng, Z.; Jacobsen, K. W. The Atomic Simulation Environment—a Python Library for Working with Atoms. *Journal of Physics: Condensed Matter* **2017**, *29* (27), 273002. https://doi.org/10.1088/1361-648X/aa680e.

(2) Horton, J. T.; Boothroyd, S.; Wagner, J.; Mitchell, J. A.; Gokey, T.; Dotson, D. L.; Behara, P. K.; Ramaswamy, V. K.; Mackey, M.; Chodera, J. D.; Anwar, J.; Mobley, D. L.; Cole, D. J. Open Force Field BespokeFit: Automating Bespoke Torsion Parametrization at Scale. *J. Chem. Inf. Model.* **2022**, *62* (22), 5622–5633. https://doi.org/10.1021/acs.jcim.2c01153.

(3) Arantes, P. R.; Sinha, S.; Palermo, G. ParametrizANI: Fast and Accessible Dihedral Parametrization for Small Molecules. *J. Chem. Inf. Model.* **2025**, *65* (20), 11357–11365. https://doi.org/10.1021/acs.jcim.5c01957.

(4) Landrum, G. RDKit: Open-Source Cheminformatics. https://www.rdkit.org (accessed 2026-08-21).

(5) O'Boyle, N. M.; Banck, M.; James, C. A.; Morley, C.; Vandermeersch, T.; Hutchison, G. R. Open Babel: An Open Chemical Toolbox. *J. Cheminform.* **2011**, *3* (1), 33. https://doi.org/10.1186/1758-2946-3-33.

(6) Batatia, I.; Kovács, D. P.; Simm, G.; Ortner, C.; Csányi, G. MACE: Higher order equivariant message passing neural networks for fast and accurate force fields. In Advances in Neural Information Processing Systems 35; Neural Information Processing Systems Foundation, Inc. (NeurIPS): San Diego, California, USA, 2022; Vol. 35, pp 11423–11436. https://doi.org/10.52202/068431-0830.

(7) Kovács, D. P.; Moore, J. H.; Browning, N. J.; Batatia, I.; Horton, J. T.; Pu, Y.; Kapil, V.; Witt, W. C.; Magdău, I.-B.; Cole, D. J.; Csányi, G. MACE-OFF: Short-Range Transferable Machine Learning Force Fields for Organic Molecules. *J. Am. Chem. Soc.* **2025**, *147* (21), 17598–17611. https://doi.org/10.1021/jacs.4c07099.

(8) Martire, S.; Decherchi, S.; Cavalli, A. OBIWAN: An Element-Wise Scalable Feed-Forward Neural Network Potential. *J. Chem. Theory Comput.* **2024**, *20* (14), 6287–6302. https://doi.org/10.1021/acs.jctc.4c00342.

(9) Wood, B. M.; Dzamba, M.; Fu, X.; Gao, M.; Shuaibi, M.; Barroso-Luque, L.; Abdelmaqsoud, K.; Gharakhanyan, V.; Kitchin, J. R.; Levine, D. S.; Michel, K.; Sriram, A.; Cohen, T.; Das, A.; Sahoo, S. J.; Rizvi, A.; Ulissi, Z. W.; Zitnick, C. L. UMA: A Family of Universal Models for Atoms. In The Thirty-ninth Annual Conference on Neural Information Processing Systems; NeurIPS, 2025.

(10) Rai, B. K.; Sresht, V.; Yang, Q.; Unwalla, R.; Tu, M.; Mathiowetz, A. M.; Bakken, G. A. TorsionNet: A Deep Neural Network to Rapidly Predict Small-Molecule Torsional Energy Profiles with the Accuracy of Quantum Mechanics. *J. Chem. Inf. Model.* **2022**, *62* (4), 785–800. https://doi.org/10.1021/acs.jcim.1c01346.
